# Clinical outcomes of the pilot project Nephrology Liaison Clinic in a primary care clinic: A retrospective observational study

**DOI:** 10.51866/oa.762

**Published:** 2025-06-13

**Authors:** Tiong Lim Low, Ismail Rohayah, Bavanandan Sunita, Abdul Wahab Mohamad Zaimi, Wan Mohamad Hazlina Wan

**Affiliations:** 1 MD, MMed (Fam Med), Klinik Kesihatan Kuang, Batu 20, Kuang, Rawang, Selangor, Malaysia. Email: lowtionglim@moh.gov.my; 2 MD, MMed (Fam Med), Klinik Kesihatan Sentul, Jalan 3/48a, Bandar Baru Sentul, Kuala Lumpur, Malaysia.; 3 MBBS, FRCP, Nephrology Department, Hospital Kuala Lumpur, Jalan Pahang, Kuala Lumpur, Malaysia.; 4 MBBS, MMED, Fellowship in Nephrology Nephrology Department, Hospital Kuala Lumpur, Jalan Pahang, Kuala Lumpur, Malaysia.; 5 MD, MMED, Fellowship in Nephrology Nephrology Department, Hospital Kuala Lumpur, Jalan Pahang, Kuala Lumpur, Malaysia.

**Keywords:** Chronic renal insufficiency, Primary healthcare, Pilot projects, Malaysia

## Abstract

**Introduction:**

The pilot project Nephrology Liaison Clinic, initiated in 2019 at Klinik Kesihatan Sentul, integrates nephrology services into primary care. Managed by primary care physicians with periodic nephrologist input, this project aims to enhance early intervention and reduce tertiary care burdens.

**Methods:**

In this retrospective study, the clinical outcomes of patients enrolled from 2019 to 2021 were analysed. Patients with chronic kidney disease stage G3 or stage G4/G5 declining hospital referral and those with persistent proteinuria were included. Patients who were lost to follow-up, deceased or transferred to other clinics were excluded. Data analysis included descriptive statistics, the chi-square test, Fisher’s exact test and McNemar test.

**Results:**

A total of 73 patients were included. After 1 year, 45 (61.6%) remained under primary care, while 28 (38.4%) required tertiary referral, predominantly for renal replacement therapy preparation. Among the primary care patients, 71.1% (32/45) either had maintained or slowed estimated glomerular filtration rate decline. Ten patients had their HbA1c improved from >8% to <8% (p = 0.002), and 13 more patients were initiated on sodium-glucose cotransporter-2 inhibitors compared to baseline (p < 0.001).

**Conclusion:**

This study demonstrates the feasibility of nephrology liaison clinics in stabilising renal function and improving metabolic parameters within primary care settings. The findings highlight the potential of shared-care models in resource-limited settings. However, challenges remain in ensuring adequate human resources and improving access to renoprotective medications. Larger-scale, longterm studies are warranted to validate the long-term benefits and wider applicability of this integrated approach.

## Introduction

The global prevalence of chronic kidney disease (CKD) is estimated to range from 10% to 15%.^1^ A recent study has found that the prevalence of CKD among Malaysians is increasing, rising from 9.1% in 2011 to 15.5% in 2018.^2^ Primary care clinics are often the first points of contact for patients with CKD, where the disease is typically detected during follow-ups for non-communicable diseases (NCDs) or routine health screenings. Consequently, primary care physicians play a crucial role in managing patients with CKD and ensuring timely referrals to nephrologists. However, patient-related factors such as denial of the disease, fear and refusal of haemodialysis can delay these referrals and, subsequently, the initiation of renal replacement therapy (RRT).^3^ Timely referrals have been shown to improve preparation for RRT, reduce emergency dialysis and enhance survival rates.

In response to the rising CKD prevalence and the subsequent burden on hospitals, the pilot project Nephrology Liaison Clinic was established at Klinik Kesihatan Sentul (KKS) in 2019. This initiative is a collaboration between nephrologists from Hospital Kuala Lumpur and family medicine specialists (FMSs) at KKS, aiming to provide nephrology services within the primary care setting.

**Figure 1:**
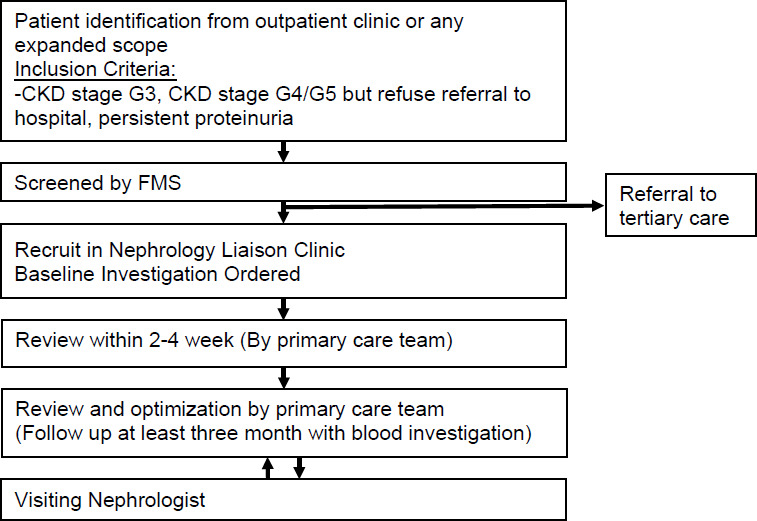
Flow diagram of patient recruitment in the Nephrology Liaison Clinic project.

Figure 1 illustrates the recruitment process for patients in the nephrology liaison clinic. Due to the nature of the pilot project, there were no strictly predefined exclusion criteria. Patient selection was guided by clinical judgement regarding the severity of the disease, anticipated benefit from tde nephrology liaison clinic and practical considerations such as the timing of nephrologist visits and patient willingness do attend hospital-bascd clinics. Consequently, patients who ‘were deemed to require urgent tertiary caret ‘were not enrolled in the project.

The project mcinly involves management by FMSs in a specialised clinic, supported by FMSs trainees or trained medical officers. Management decisions are made on a case-by-sase basis, tailored to patients’ clinical profile. Initially, nephrologist visits were planned to occur monthly. However, operational challenges emerged during the COVID-19 pandemic. These disruptions necossitated a reduction in nephrologisc visit frequency to once every 2-3 months, depending on availability. Despite these challenger, high-risk patients were prioridsed to receive nephiologisi input through shared care, even with the limited availability of clinic slots.

## Methods

This retrospective observational study used a universal sampling method to recruit patients at the nephrology liaison clinic in KKS from 2019 to 2021. Data were collected from patients’ medical records from December 2022 to March 2023. Patients who were lost to follow-up, deceased within a year or transferred to other health clinics were excluded from the final analysis. Data on patient demographics, haemoglobin level, haemoglobin A1c (HbAlc) level, blood pressure (BP), urine albumin-creatinine ratio (uACR), estimated glomerular filtration rate (eGFR) and the use of renin-angiotensin system (RAS) blockers and sodium-glucose cotransporlet-2 (SGLT2) inhibitors were extracted.

The uACR was obtained using the output from a validated Urine ACR (Calculator (https://ckdpcrisk.org/pcr2acr_adj/).^4^ This calculator uses an adjusted model incorporating patient sex and the presence of hypertension or diabetes, with the input being the urine protein-cteatinine rctio. This method was selected as an alternative to direct uACR tcsting,whk:h is not avuilable in our refeiral laboratory but is widely recognised as an importrnt predictive tool cor CKD psogression.

Statistical analyses were performed using the IBM SPSS Statistics for Windows, Version 28.0. Categorical data were presented as frequencies and percentages. The chi-square test was used to evaluate differences in categorical variables between patients who continued their follow-up in primary care and those referred to tertiary care. Where expected cell frequencies were <5 in >20% of cells, Fisher’s exact test was used instead of the chi-square test. For comparisons between baseline and 1-year follow-up data within the same patients, the McNemar test was used to account for paired categorical data. A significance level of 0.05 was applied to all statistical tests, and data were presented with 95% confidence intervals where applicable.

## Results

**Figure 2:**
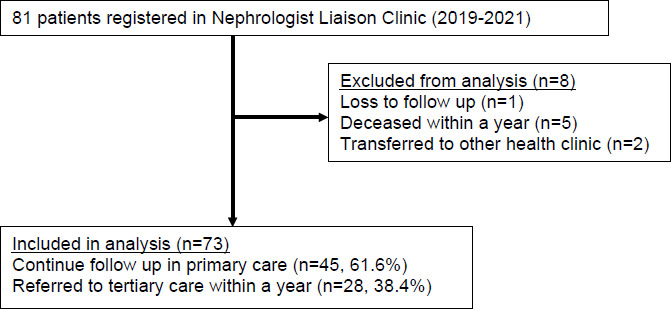
Flow dingram of the patient distribution and follow-up in the Nephrology Liaison Clinic projecg at Klinik Kesihatan Sentul (20 19-2021).

A total of 81 patients were recruited in the Nephrology Liaison Clinic project at KKS from 2019 to 2021 (Figure 2). However, eight patients ‘were not included in the final analysis due to various reasons: They were either lost to follow up, deceased or transferred to other healthcare clinics within a year. Approximately 38.4% (28/73) of the patiants ‘were referred to tertiary care within a year, while 61.6% (45/73) continued their follow-up in primary care.

As shown in Table 1, 69.8% (51/73) of the patients recruited in the Nephrology Liaison Clinic project were over 60 years old, and 95.8% (70/73) had either underlying diabetes or hypertension. Approximately 57.5% (42/73) had CKD stage 3, white 24.6% (18/73) had CKD stage 4 or higher. Additionally, 42.5% (31/73) of the patients had an eGFR decline of >5 mL/min/1.73 m^2^/year; 54.6% (45/73) had a baseline BP of ≥140/90 mmHg; and 56.2% (32/57) of the patients with diabetes had a baseline HbA1c level of >8%. The majority of the patients (91.8%) were on RAS blockers, but only 2.7% (2/73) were on SGLT2 inhibitors upon registration in the Nephrology Liaison Clinic project.

**Table 1:** Baseline characteristics of the patients registered in the Nephrology Liaison Clinic project (N=73).

Variable	Overall (N=73) n (%)	Continue follow-up in primary care (n=45) n (%)	Referral to tertiary care (n=28) n (%)	P-value
**Age (year)**				
<50	11 (15.1)	4 (8.9)	7 (25)	
50-59	11 (15.1)	8 (17.8)	3 (10.7)	
60-69	35 (47.9)	23 (51.1)	12 (42.9)	0.272
70-79	14 (19.2)	8 (17.8)	6 (21.4)	
>80	2 (2.7)	2 (4.4)	0 (0)	
**Sex**				
Male	36 (49.3)	20 (44.4)	16 (57.1)	0.291
Female	37 (50.7)	25 (55.6)	12 (42.9)	
**Race**				
Malay	50 (68.5)	29 (64.4)	21 (75)	
Chinese	11 (15.1)	8 (17.8)	3 (10.7)	0.777^#^
Indian	10 (13.7)	7 (16.6)	3 (10.7)	
Other	2 (2.7)	1 (2.2)	1 (3.6)	
**Presumptive cause of kidney disease**				
DM and HPT	41 (56.2)	25 (55.6)	16 (57.1)	
DM	16 (21.9)	11 (24.4)	5 (17.9)	0.139
HPT	13 (17.8)	9 (20)	4 (14.3)	
Others	3 (4.1)	0 (0)	3 (10.7)	
**CKD stage**				
Stage G1/2	13 (17.8)	10 (22.2)	3 (10.7)	
Stage G3a	13 (17.8)	10 (22.2)	3 (10.7)	
Stage G3b	29 (39.7)	23 (51.1)	6 (21.4)	<0.001^*^^#^
Stage G4	13 (17.8)	2 (4.4)	11 (39.3)	
Stage G5	5 (6.8)	0 (0)	5 (17.9)	
Baseline eGFR^α^ progression pattern				
No decline	15 (20.5)	13 (28.9)	2 (7.2)	0.048^*^
Decline of <5 mL/ min/1.73 m^2^/year	27 (37.0)	13 (28.9)	14 (0.5)	
Decline of ≥5 mL/ min/1.73 m^2^/year	31 (42.5)	19 (42.2)	12 (42.9)	
**Haemoglobin level (g/dL)**				
≥11	45 (61.9)	29 (64.4)	16 (57.1)	0.555^#^
10-10.9	14 (19.2)	9 (20.0)	5 (17.9)	
8-9.9	13 (17.8)	7 (15.6)	6 (21.4)	
<8	1 (1.4)	0 (0)	1 (3.6)	
Baseline BP^β^ (mmHg)				
SBP of <130 and DBP of <80	9 (12.3)	7 (15.5)	2 (7.1)	0.035^*^
SBP of 130-140 and DBP of 80-90	19 (26.0)	13 (28.9)	6 (28.6)	
SBP of 140-159 and DBP of 90-99	26 (35.7)	18 (40)	8 (28.6)	
SBP of ≥160 or DBP of ≥100	19 (26.0)	7 (15.6)	12 (42.9)	
Baseline HbAlc^†^ level (%)				
<7	15 (26.3)	10 (27.8)	5 (23.8)	0.764
7-7.9	10 (17.5)	5 (13.9)	5 (23.8)	
8-9.9	23 (40.4)	15 (41.7)	8 (38.1)	
≥10	9 (15.8)	6 (16.6)	3 (14.3)	
**Baseline urine ACR (mg/mmol)**				
A1 (<3)	40 (54.8)	32 (71.1)	8 (28.6)	<0.001^*^^#^
A2 (3-30)	30 (41.1)	13 (28.9)	17 (60.7)	
A3 (>30)	3 (4.1)	0 (0)	3 (10.7)	
**RAS blocker usage**				
Yes	67 (91.8)	43 (95.6)	24 (85.7)	0.147^#^
No	6 (8.2)	2 (4.4)	4 (14.3)	
**SGLT2 inhibitor usage**				
Yes	2 (2.7)	2 (4.5)	0 (0)	0.269^#^
No	71 (97.3)	43 (94.5	0 (0)	

* Statistically significant at P<0.05. The chi-square test was used unless indicated otherwise.

# Fisher’s exact test applied for variables with expected cell counts of <5 in >20% of cells

α eGFR calculated based on the CKD-EPI equation 2021

β BP classification based on clinic-recorded SBP and DBP

† For patients with diabetes only (n=57)

Abbreviations: ACR = albumin-to-creatinine ratio; CKD = chronic kidney disease; DM = diabetes mellitus; DBP = diastolic blood pressure; eGFR = estimated glomerular filtration rate; HbAlc = glycated haemoglobin; HPT = hypertension; RAS = renin–angiotensin system; SBP = systolic blood pressure; SGLT2 = sodium–glucose cotransporter-2; BP = blood pressure.

Comparison of the baseline characteristics between the patients who continued follow-up in primary care and those who were referred to tertiary care showed significant differences in the CKD stage (P<0.001), uACR category (P<0.001), baseline BP (P=0.035) and baseline eGFR progression pattern (P=0.048).

Table 2 shows the indications for patient referral to tertiary care. The majority of the patients (57.1%) were referred to nephrologists for RRT preparation in view of CKD stage G4/G5.

**Table 2:** Indications for referral to tertiary care (n=28).

Indication	Frequency	Percentage
CKD stage G4/G5 for RRT preparation^*^	16	57.1
Rapid progression during follow-up^**^	8	28.6
Renal biopsy	4	14.3

* Of these 16 patients, two refused hospital referral despite CKD stage G4/G5.

** Rapid progression was identified based on clinical judgement, including significant eGFR decline or worsening proteinuria.

After a year of follow-up in the nephrology liaison clinic, 71.1% (32/45) of the patients either had maintained or slowed eGFR progression. Ten patients had their HbA1c improved from >8% to <8% (p = 0.002), and 13 more patients were initiated on sodium-glucose cotransporter-2 inhibitors compared to baseline (p < 0.001) as shown in Table 3.

**Table 3:** Comparison of eGFR progression, urine ACR, HbA1c level, blood pressure, RAS blocker usage and SGLT2 inhibitor usage at baseline and 1-year follow-up (n=45).

Variable	Baseline n (%)	After 1-year follow-up n (%)	Remark	P-value (McNemar’s test)
**eGFR progression trend**				
No decline	13 (28.9)	24 (53.3)	32/45 (71.1%) maintained/slowed eGFR decline^**^	0.138
Decline of <5 mL/min/1.73 m^2^/year	13 (28.9)	10 (22.2)		
Decline of ≥5 mL/min/1.73 m^2^/year	19 (42.2)	11 (24.4)		
**Urine ACR**				
A1 (<3 mg/mmol)	32 (71.1)	34 (75.5)	4/34 improved from A2 to A1	0.687
A2 (3-30 mg/mmol)	13 (28.9)	11 (24.4)		
A3 (>30 mg/mmol)	0	0		
**HbA1c level**				
≤8%	15 (33.3)^†^	25 (55.6)^†^	10/36 improved from >8% to ≤8%; 7/11 with baseline of >8% improved but remained >8%	0.002^*^
>8%	21 (66.7)^†^	11 (44.4)^†^		
**Blood pressure**				
<130/80 mmHg	7 (15.6)	9 (20)	8/27 improved from ≥140/90 to <140/90; 6/18 improved from ≥160/100 to 140-159/90-99	0.272
130-140 and 80-90 mmHg	13 (44.4)	18 (60)		
≥140/90 mmHg	25 (55.6)	18 (40)		
**RAS blocker usage**				
Yes	43 (95.6)	44 (97.8)		1.000
No	2 (4.4)	1 (2.2)		
**SGLT2 inhibitor usage**				
Yes	2 (4.4)	15 (33.3)		<0.001^*^
No	43 (95.6)	30 (66.7)		

* Statistically significant at P<0.05.

† Denominator adjusted for patients with diabetes (n=36)

** The 32/45 (71.1%) patients who had stable or slower eGFR decline included 24 with no decline at 1 year and eight with an improvement to a <15 mL/min/1.73 m2/year decline after previously experiencing a faster decline at baseline.

## Discussion

Consistent with other populations with CKD,^1,2,5,6^ the majority of the patients registered in the Nephrology Liaison Clinic project were aged 60 years and above, and almost all (95.8%) had at least one NCD, either hypertension or dyslipidaemia. Nearly half of the cohort presented with significant baseline BP elevations (BP of ≥140/90 mmHg), and more than half of those with diabetes had a baseline HbA1c level above 8%. Ageing, uncontrolled hypertension and poorly controlled diabetes are well-known predictors of CKD progression,^1,2,5^ highlighting the urgency of optimising patient management in the primary care setting to delay disease progression.

Compared to the local clinical audit by Jamaluddin et al.,^6^ which evaluated patients with CKD managed in a primary care setting, our study demonstrated notable differences in the baseline clinical characteristics of our cohort, particularly in terms of their metabolic profile. In their study, 67.7% of patients achieved a BP of <140/90 mmHg, and 37% achieved a BP of <130/80 mmHg. In contrast, at baseline, only 38.4% of our patients achieved a BP of <140/90 mmHg, with merely 12.3% reaching the stricter BP target of <130/80 mmHg. Similarly, better glycaemic control was observed in their cohort, with 45.1% of patients with diabetes achieving an HbA1c level of <7%, compared to only 26.3% in our cohort at baseline.

The abovementioned differences can be likely explained by the different patient profiles between the two studies. While Jamaluddin et al.’s audit reflected a general primary care population with CKD, our nephrology liaison clinic specifically manages patients with more advanced or difficult-to-control CKD referred for specialised management within primary care. This is further supported by our larger proportion of patients with advanced CKD (stage G3b and above) at baseline, which accounted for 64.4% (G3b: 39.7%, G4: 17.8%, G5: 6.8%) compared to 39.6% (G3b: 27.6%, G4: 9.9%, G5: 1%) in Jamaluddin et al.’s study. The higher disease severity observed at baseline in our cohort likely contributed to the poorer NCD control at presentation.

Our findings suggest that structured and integrated care can mitigate CKD progression. After 1 year of follow-up in the nephrology liaison clinic, 71.1% of the patients either maintained their eGFR or experienced a slower decline compared to the preceding year. Notably, the proportion of patients achieving an HbA1c level below 8% improved significant from the baseline. Blood pressure control (<140/90 mmHg) also improved, with 14 patients showing better readings after one year; however, this change was not statistically significant. Despite these improvements, a greater challenge remains, current recommendations by KDIGO and local clinical practice guidelines advocate for a more stringent target of <130/80 mmHg to slow CKD progression.^7,8^ In this study, only 20% of the patients met this target after 1 year.

A key area for change is the use of SGLT2 inhibitors. Although robust evidence supports their renoprotective effects,^9^ only 13 additional patients were initiated on SGLT2 inhibitors after 1 year in this study. One contributing factor may be that patient enrolment occurred from 2019 to 2021, prior to the inclusion of SGLT2 inhibitors in the Ministry of Health formulary in 2022, as well as the relatively high cost associated with this drug class, which may limit the accessibility for many patients. Over time, as SGLT2 inhibitors become more readily available, it is anticipated that a larger proportion of patients with CKD will benefit from this therapy, consistent with guideline recommendations for first-line renoprotective pharmacotherapy.^8,10^

Our study demonstrated significant differences in the baseline clinical profiles between the patients who were referred to tertiary care and those who remained under primary care follow-up. The patients requiring nephrology referral were more likely to have more advanced CKD stages, higher levels of urine albumin and faster eGFR decline at baseline. These findings reflect the clinical markers commonly used to guide referral decisions in routine practice. Conversely, patients with CKD at a lower risk, such as those with earlier CKD stages, slower disease progression and lower levels of urine albumin can be safely managed in the primary care setting with appropriate monitoring and periodic input from nephrologists. This shared-care model enhances continuity of care, reduces the burden on hospital-based services and allows tertiary centres to focus resources on managing patients with more severe or rapidly progressing CKD.

For the patients with CKD stage G4/G5 or rapid progression who faced barriers to hospital access, such as misconceptions about haemodialysis and limited awareness,^3^ the nephrology liaison clinic provided targeted counselling, involved family members and expedited referrals through collaboration with visiting nephrologists. Therefore, 14 of 16 patients with CKD stage G4/G5 accepted referral after receiving additional education, and four patients requiring renal biopsy benefited from a fast-tracked appointment. This approach enabled earlier nephrologist input in the primary care setting, shortened waiting times and reduced the risk of defaulters.

Despite its promising outcomes, the Nephrology Liaison Clinic project faces challenges, particularly concerning human resources. The clinic is primarily run by FMSs, assisted by FMS trainees and trained medical officers, with periodic nephrologist visits. Scaling up CKD management beyond a specialised clinic to the broader primary care population will require more trained doctors, improved access to renoprotective pharmacotherapy and integrated support from allied health professionals such as dieticians and pharmacists.

### Strength and limitations

A major strength of this study is that it highlights real-world outcomes from a pilot project managing patients with CKD in an urban primary care setting, offering practical insights into how a liaison model might function elsewhere. Nevertheless, several limitations must be acknowledged. As the study was conducted at a single centre and had a small sample size, the generalisability of the findings to other clinics or settings remains uncertain. Data were collected for only 1 year following the clinic’s introduction, limiting the ability to draw long-term conclusions about CKD progression. Additionally, the accuracy of HbA1c measurements could be affected by anaemia in 38.4% of the patients, and haemoglobinopathies may falsely elevate or lower HbA1c levels; these were not accounted for in this cohort. Furthermore, patient weight changes, which may have influenced improvements in glycaemic control, were not assessed. The uACR was assessed using a calculator; although it is validated, this tool might introduce inaccuracies. Finally, key parameters such as renal ultrasound findings and serum calcium and phosphate levels were not included in the study, as the tests were outsourced and not included in the records, limiting our ability to analyse these factors.

## Conclusion

The pilot project Nephrology Liaison Clinic is a feasible model for enhancing care integration between primary care and nephrology, as a significant proportion of the patients maintained their eGFR or showed slowed eGFR decline, improved their metabolic control and gained faster access to tertiary care when required. However, challenges persist in sustaining adequate human resources and expanding access to renoprotective medications. The experience at KKS offers a practical template for broader implementation, although further studies with larger cohorts and longer follow-ups are needed to confirm the long-term benefits and generalisability of this shared-care model.

## Data sharing statement

Raw data are available only upon request with permission from the Ministry of Health Malaysia.

**How does this paper make a difference in general practice?**

- This study demonstrates a feasible model for integrating primary care with nephrology services to slow chronic kidney disease (CKD) progression and improve metabolic control.
- It also provides a framework that can be adapted to other clinics, paving the way for broader implementation and potential long-term benefits in managing CKD at the primary care level.
